# Effect of beta-agonist type and timing of Experior feeding on calculated cumulative ammonia gas emissions, live growth performance, and carcass outcomes, and objective tenderness outcomes of feedlot steers

**DOI:** 10.1093/tas/txaf009

**Published:** 2025-01-24

**Authors:** Wilsey M Wendler, Michael S Davis, Walter C Koers, Phillip J Rincker, Nathan A Pyatt, Loni W Lucherk, Ty E Lawrence

**Affiliations:** Beef Carcass Research Center, Department of Agricultural Sciences, West Texas A&M University, Canyon, TX 79016, USA; Bos Technica Research Services, Salina, KS 67401, USA; Bos Technica Research Services, Salina, KS 67401, USA; Elanco Animal Health, Greenfield, IN 46140, USA; Elanco Animal Health, Greenfield, IN 46140, USA; Beef Carcass Research Center, Department of Agricultural Sciences, West Texas A&M University, Canyon, TX 79016, USA; Beef Carcass Research Center, Department of Agricultural Sciences, West Texas A&M University, Canyon, TX 79016, USA

**Keywords:** antagonist, cattle, days on feed, feed efficiency, lubabegron fumarate

## Abstract

Our objective was to compare beta-agonist feeding strategies and evaluate Experior (EXP) with added days on feed (DOF) for live growth performance and carcass outcomes. Steers (n = 2,517; initial BW = 336 ± 23 kg) were allocated to 36 pens in a randomized, complete-block design and assigned to 1 of 6 treatments. Treatments were negative control (no beta-ligand; CON), Optaflexx (ractopamine hydrochloride, 300 mg·head^−1^·day^−1^ for 35 d; OPT), and Experior (lubabegron fumarate, 36 mg·head^−1^·day^−1^ for 56 d + 4 d removal; 0EXP) with all three treatments fed for 198 d. Remaining treatments were fed EXP for same duration as 0EXP, but total DOF were adjusted by −14 d (−14EXP), +14 d (+14EXP) and +28 d (+28EXP). Statistical analyses were conducted using mixed models; treatment was the fixed effect, block was a random effect, means were separated with the Tukey-Kramer adjustment, and contrasts were calculated to test linear or quadratic effects of EXP across DOF. No treatment differences (*P* ≥ 0.72) were observed for daily dry matter intake. Calculated cumulative ammonia (NH_3_) gas emissions (CCAGE) expressed either as total NH_3_ emitted (g) or NH_3_ emitted per kg of BW or HCW was reduced (*P* < 0.01) by 5.6–8.5%, 5.1–10.7%, and 6.3–13.6%, respectively for 0EXP, +14EXP and +28EXP compared to CON. Carcass ADG of 0EXP steers was 6.8% greater (*P* < 0.01) than CON, which had similar (*P* ≥ 0.10) ADG to OPT, +14EXP, and +28EXP steers. Carcass G:F of 0EXP steers was improved 5.0% compared to CON (*P* = 0.01) and was not different (*P* ≥ 0.10) from OPT, −14EXP or +14EXP steers. Hot carcass weight of 0EXP, +14EXP, or +28EXP steers was 14–37 kg heavier (*P* < 0.01) than CON, which did not differ (*P* = 0.81) from OPT. Steers fed EXP had 1.15–2.5% point increase (*P* < 0.01) in dressed yields. *Longissimus* muscle area was 4.03–6.23 cm^2^ larger (*P* < 0.01) in steers fed EXP compared to CON, which tended to differ (*P* = 0.08) from OPT. Marbling score was 31–39 points lower (*P* ≤ 0.02) for −14EXP compared to CON and OPT, whereas 0EXP tended (*P* = 0.09) to differ from CON and +14EXP and +28EXP were similar (*P* ≥ 0.28) to CON and OPT. Slice shear force values were 20.1% greater (*P* < 0.02) for +14EXP compared to CON, whereas 0EXP tended (*P* = 0.09) to be greater and +28EXP, −14EXP and OPT were similar (*P* ≥ 0.28) to CON. This study illustrates EXP decreased CCAGE and increased HCW with minimal quality changes when fed the last 56 d on feed.

## Teaser

At the same days on feed, Experior supplementation compared to positive control (ractopamine hydrochloride; OPT) or negative control (no beta agonist; CON), increased carcass growth performance, hot carcass weight and lowered quality grade distribution. Experior did not show significant advantages in live growth performance, such as live body weight, average daily gain, or gain:feed.

## INTRODUCTION

Experior (EXP; lubabegron fumarate), a β1 - β2—adrenergic antagonist/β3-adrenergic agonist, has been approved by the US Food and Drug Administration (FDA) for the reduction of NH_3_ gas emissions per kilogram of live body weight (BW) and hot carcass weight (HCW) in steers and heifers fed in confinement for slaughter during the last 14–91 d on feed (FDA-FOI, 2018). Because of its dual modulating blocking, and activating effect, lubabegron is most appropriately classified as a β modulator. Additionally, EXP supplementation has demonstrated improved growth performance, and carcass attributes by increasing average daily gain (ADG), gain to feed, final BW, HCW, carcass yield and longissimus muscle area (LMA; Kube et al., 2021; Teeter et al., 2021). Clinical effectiveness studies have documented a 16% reduction in NH_3_ gas emissions per unit of HCW of when lubabegron was fed at 5.5 mg/kg of DM for 91 d to steers (FDA-FOI, 2018). Limited research has investigated LMA changes concomitant with marbling expression while feeding EXP. Additionally, limited research is available that evaluates the effect of EXP compared to a different beta agonist feeding strategy. Previous research pertaining to EXP has evaluated the dosage and duration of lubabegron fed to beef steers. Throughout multiple small and large pen studies (Kube et al., 2021; Teeter et al., 2021), the recommendation of 36 mg·head^−1^·day^−1^ for 28–63 d has been reported as the most beneficial to improve gain efficiency and minimize negative impacts upon marbling score. There have been few studies comparing both the effects of Optaflexx and EXP on growth performance and carcass characteristics. More recently, McAtee et al. (2024) reported EXP reduced gas emissions per kg of HCW by 6.7% in the finishing phase. The purpose of this study was to assess live growth performance and carcass outcomes by evaluating varying days on feed (DOF) prior to initiation of EXP, while keeping the dosage and duration of EXP constant.

## MATERIALS AND METHODS

The experimental phase of the trial was initiated in July 2022 and completed in March 2023, at a large-pen commercial feedlot in Oklahoma. All study procedures were reviewed and approved by Institutional Animal Care and Use Committee (Approval number EIAC-1783).

### Experimental Design

Steers (n = 2,517; initial BW = 336 ± 23 kg) were allocated to 36 pens (~70 hd/pen) in a randomized, complete block design and assigned to 1 of 6 treatments with pen as the experimental unit. Treatments were negative control (no beta-ligand; CON), positive control—Optaflexx (ractopamine hydrochloride, 300 mg·head^−1^·day^−1^ for 35 d; OPT), and Experior (lubabegron fumarate, 36 mg·head^−1^·day^−1^ for 56 d + 4 d removal; 0EXP) with all three treatments fed for 198 d. Remaining treatments were fed EXP for same duration as 0EXP, but total DOF were adjusted by −14 d (−14EXP), +14 d (+14EXP) and +28 d (+28EXP) resulting in total DOF being 184, 212, and 226 d. The feeding duration of 35 d was selected for OPT, due to the current industry recommendation being 35 d over 28 or 42 d. Bittner et al. (2016) observed the response curve of Optaflexx duration with peak timing being near 5 wk and some diminishment at 42d.

Each of the six blocks contained six treatments (~420 hd/treatment). Pens assigned to +14EXP were fed an additional 2 d due to scheduling logistics at the harvest facility. The study was powered based on a denominator variable (i.e., HCW and quality grade) because the numerator was a calculated value and the denominator was based on actual animal or carcass weight. A minimum of six pen replicates were needed to detect a 4.5 kg HCW treatment difference or greater and conduct linear and quadratic contrasts among compositional endpoints with expected 3–5% differences in USDA Choice quality grade.

### Live Cattle Procedures

British and European crossbred steers were sourced from stocker operations in Oklahoma, Missouri and Arkansas. Phenotypically, steers were predominately black-hided (65%) and medium to large in frame size. The live animal portion of the trial was conducted by Bos Technica Research Services and steers were received at Xcel Feedyard in Watonga, OK beginning in July 2022. Upon arrival, steers were kept separate based on source and arrival date until a satisfactory number were present to meet the head count criteria for a single statistical block. Later, steers were randomly assigned to treatment pens within a block before the initiation of the study phase. Pre-study screening occurred prior to randomization to exclude those with abnormal health and performance while allowing for a uniform body weight for animals enrolled to the study. A qualified evaluator examined and screened steers for eligibility based on the following inclusion criteria: confirmed to be a steer and determined to be in good health. Exclusion criteria included heifers, animals that appeared unthrifty, ill, injured or were > 2 standard deviations of mean BW.

Immediately following randomization, steers were weighed by pen on a 21.34 m platform scale (Fairbanks TRBT88, Kansas City, MO) in one draft to determine starting weights; mean BW of the six blocks was 335.66 kg. Minimum and maximum within-block BW across all six blocks were 323.87 and 347.00 kg, respectively. Study initiation dates ranged from July 21, 2022 to July 29, 2022 for the six blocks.

Initial processing procedures included recording of individual BW, placement of a colored numbered ear tag, vaccination, treatment with an internal and external parasiticide, and administration of a growth promoting implant. Steers were vaccinated against clostridial pathogens with Vision^®^ 7 (Merck Animal Health, Rahway, NJ) and against bovine respiratory disease pathogens with viral Titanium^®^ 3 (Elanco Animal Health, Greenfield, IN). Anthelmintics used for internal and external parasites were Dectomax^®^ (10 mg doramectin · 49.9 kg^−1^ BW; Zoetis Animal Health, Parsippany, NJ) and Safeguard^®^ (5 mg fenbendazole · kg^−1^ BW; Merck Animal Health). Steers were initially implanted at day 0 with Component TE-IS with Tylan (80 mg trenbolone acetate + 16 mg estradiol + 29 mg tylosin tartrate; Elanco Animal Health) and were reimplanted 82–84 d prior to harvest with Component TE-200 with Tylan (200 mg trenbolone acetate + 20 mg estradiol + 29 mg tylosin tartrate; Elanco Animal Health).

### Ammonia Gas Emissions

Cumulative NH_3_ gas emissions were calculated from (Brown et al., 2019):


$$\begin{array}{l} \begin{array}{lllllll} & y\left[log_{10}\left(cumulative\;NH_{3}gas\;emissions,\;g\cdot animal^{-1}\right)\right]=\; & 0.06758372\times \left(cumulative\;N\;intake,\;kg\cdot animal^{-1}\right)\; & -0.011425\times \left(lubabegron\;dose,\;g\cdot ton^{-1}\right)\; & -4.9743281\times \left(1/cumulative\;duration,\;d\right)-0.0012361\; & \times {\left(cumulative\;N\;intake,\;kg\cdot animal^{-1}\right)}^{2}\; & +0.0002744\times {\left(outdoor\;temperature\;{,}^{\circ }C\right)}^{2}+3.01996229\; \end{array} \end{array}$$

where: lubabegron dose was based on a 100% DM diet. Cumulative N intake per animal was calculated as the sum of weekly average feed consumed, adjusted for the number of animals in each pen each day × (assayed crude protein [CP, %] ÷ 6.25) from weekly feed samples collected during the treatment period. The ratio of calculated cumulative NH_3_ gas emissions (CCAGE) to final BW and HCW (kg) was calculated for the 56 d Experior treatment period. Outdoor temperature was calculated as the average of the daily mean ambient temperature documented at Watonga, Oklahoma during the treatment period. The CCAGE were determined by CCAGE·BW^−1^ (g·kg^−1^) and CCAGE·HCW^−1^ (g·kg^−1^), the primary outcomes of interest. Calculations for OPT treatment were omitted and not used in the calculated equation.

### Health Observations

Cattle were observed by trained personnel who rode pens horseback daily to identify animals requiring veterinary care, particularly for respiratory issues or other ailments including digestive and mechanical distress. Abnormal health observations were recorded even if considered normal for feedlot cattle. Health observations requiring an animal to be classified as “removed” or “rejected” were abnormalities the observer considered as resulting in pain or distress to the animal, or likely to result in further deterioration of the animal’s health. Animals removed from the study during the treatment phase were penned separately. Decisions to remove an animal from treatment phase were made by the investigator or manager.

Once animals needing treatment were identified, those animals were removed from pens and walked to the treatment facility, where appropriate treatment was administered based on the diagnosis. Animals with respiratory disease were treated according to the following protocol: first pulls received Resflor^®^ (40 mg florfenicol · kg^−1^ BW; 2.2 mg flunixin · kg^−1^ BW; Merck Animal Health), second pulls received Excede^®^ (6.6 mg ceftiofur crystalline free acid · kg^−1^ BW; Zoetis Animal Health), and third pulls received Draxxin^®^ (2.5 mg tulathromycin · kg^−1^ BW; Zoetis Animal Health). Post-treatment intervals (PTI) between pulls were 5, 7 and 10 d, respectively. Any animal presented for respiratory treatment following Draxxin PTI was removed from the study.

Weights were documented for each animal undergoing respiratory treatment, utilizing the same equipment employed for recording individual weights during processing. Rectal temperatures were measured using an AG Medix Model AG-102 (Mukwonago, WI) thermometer.

### Diet Formulation and Feed Assays

Steers were fed a diet containing monensin sodium (Rumensin^®^ 90, Elanco) which was included in all rations with concentration increasing from 25 g of monensin · 907.4 kg^−1^ of DM in Ration 1–44.4 g of monensin · 907.4 kg^−1^ of DM in Ration 4. Tylosin phosphate (Tylan^®^ 100, Elanco) was included in all rations to provide 90 mg of tylosin·steer^−1^·d^−1^. Steers were transitioned from a basal finishing ration to the respective treatment diet for the final feeding period. Cattle in EXP treatments were switched from the basal finishing ration to the finishing ration containing the appropriate concentration of lubabegron Type A premix at the start of the treatment period. Table 1 lists composition and formulated nutrient content of the finishing diet, which was designed to meet or exceed the minimum nutrient requirements cited in Nutrient Requirements of Beef Cattle (NASEM, 2016).

**Table 1.** Dietary ingredient composition (DM, %) of experimental rations.

Item	Ration 1	Ration 2	Ration 3	Ration 4	Ration 4
*Ingredient, % dry matter*					
Steam-flaked corn	52.9	62.7	76.7	86.5	86.5
Alfalfa hay	35.9	25.6	10.5	2.7	2.7
Sorghum silage	0.0	0.0	2.2	3.3	3.3
Cane molasses	5.8	5.0	2.6	0.0	0.0
Fat	0.0	1.3	1.3	1.3	1.3
Starter supplement^a^	5.4	5.4	6.7	0.0	0.0
Finisher supplement^b^	0.0	0.0	0.0	6.2	6.2
Feed additive^c^	0.0	0.0	0.0	0.0	0.0
*Formulated nutrient analysis, %*					
Moisture	22.04	21.85	23.78	24.68	24.31
Crude protein	13.72	12.83	12.19	12.57	12.68
Non-protein nitrogen	1.61	1.61	2.02	2.61	2.62
Crude fiber	14.30	11.00	7.14	4.90	4.91
Fat	2.83	4.37	4.90	5.25	4.98
Calcium	0.99	0.84	0.72	0.67	0.68
Phosphorus	0.27	0.27	0.28	0.29	0.29
Potassium	1.50	1.24	0.83	0.64	0.65
Salt	0.21	0.21	0.27	0.28	0.28

^a^Contained at least 45% crude protein and no more than 30% non-protein nitrogen.

^b^Contained at least 56% crude protein and no more than 42% non-protein nitrogen.

^c^Supplied monensin sodium (Rumensin 90, Elanco) at levels of 25, 32, 44.4 g/907.4 kg DM in Ration 1, 2, 3, and 4, respectively. Supplied tylosin phosphate (Tylan 100, Elanco) in all rations at 90 mg/animal daily. Optaflexx was included last 35 d prior to harvest according to treatment (300 mg/animal daily). Experior was included according to treatment 60 d prior to harvest (3.2 g/907.4 kg of DM) and withdrawn from ration 4 d prior to harvest.

All type C feeds containing the appropriate concentrations of EXP and OPT were prepared at the study site by weighing ingredients on an analytical platform scale (Acculab, Model ALC 2100.2 Goettingen, Germany) to the nearest 0.01 g. Sixty days prior to slaughter, type A premix was added to the basal diet to provide either 0.0 (CON) or 3.2 g·ton^−1^ (DM basis) EXP in a type C medicated feed. The 1% Type A premix was added into the ration by using a flush bowl system whereby the medicated article was mixed with water and discharged from the mixing vessel with a pump. Additives were mixed in a flush bowl stirred with 19 liters of water for 45 s. The resulting mixture was subsequently flushed onto the feed truck. Immediately following discharge onto truck, the flush bowl was rinsed with an additional 22 liters of water and discharged into the truck. Before blending CON treatment batches, the truck-mounted mixer was flushed with silage to ensure there was no carryover of the test compound.

Analysis of monensin, ractopamine and lubabegron was conducted by Eurofins Animal Testing (Indianapolis, IN). Samples for Rumensin were collected daily and composited every 2 wk prior to analysis, whereas during treatment feeding of EXP and OPT, ration samples were collected from a single load of feed each week and sent overnight for analysis of ractopamine and lubabegron levels. Dry matter was determined daily for all rations by weighing a 100-g sample into a pan using a digital scale (Acculab, Model ALC 2100.2 Goettingen, Germany). Samples were placed into a forced air oven (VWR Scientific—1350FD Cornelius, OR) for 24 h at 100 °C. After 24-h of drying, samples were re-weighed to determine DM content.

Ration samples for nutrient analysis were collected daily and composited every 2 wk prior to submission to SDK Laboratory (Hutchinson, KS). The diet was assayed for moisture, crude protein (CP), non-protein nitrogen (NPN), crude fiber, fat (ether extract), calcium (Ca), phosphorus (P), potassium (K), and sulfur (S).

### Feeding Procedures

Treatment diets were fed for ad libitum consumption. Additionally, water was provided ad libitum throughout the entire study duration. A step-up ration system involving two intermediate diets was used while increasing concentrate levels. Throughout the feeding period, steers were transitioned from the starter diet to the appropriate finishing diet by utilizing three step-up diets fed a minimum of 5 d each until steers were solely fed the finisher diet. By d 21 all cattle were on the finishing diet. Steers were fed three times daily using a single-axle, International truck equipped with a Harsh 575H mixer/delivery box. During periods of inclement weather (i.e., rain, snow) or at the discretion of the site manager, feed remaining in the bunk prior to the first feeding was removed, sampled, weighed back, and then discarded by composting. Weighback samples were analyzed for DM using procedures described previously. Total DM weighed back from each pen was then calculated and subtracted from DM offered the day feed was originally fed. Cattle were switched from the basal finishing ration to the respective treatment diets containing either OPT, EXP, or CON treatment. Experior feeding started in November 2022, December 2022, and January 2023 for each corresponding treatment. On the day prior to obtaining final weight, steers were fed 80% of the recent 5-d average as-fed intake to lessen gut fill. Each pen of steers was weighed in two drafts (25–40 steers/draft) and placed in holding pens. A 4% pencil shrink was applied to all final pen weights.

### Animal Mobility

Animal mobility was assessed after antemortem inspection immediately prior to steers entering the harvest floor. The trained mobility scorer used the North American Meat Institute’s (NAMI) mobility scoring system (Edwards-Callaway et al., 2017) to evaluate cattle mobility. The NAMI mobility scoring system uses a 4-point scale where 1 = normal, walks easily with no apparent lameness or change in gait; 2 = keeps up with normal cattle when the group is walking and exhibits one or more of the following: stiffness, shortened stride, or slight limp; 3 = lags behind normal cattle when the group is walking, exhibits one or more of the following: obvious stiffness, difficulty taking steps, obvious limp, or discomfort; 4 = extremely reluctant to move, even when encouraged by handlers.

### Slaughter and Carcass Characteristics

Final weights of steers were obtained on the day of harvest (January 24^th^, February 7^th^, February 8^th^, February 23^rd^, and March 7^th^). Steers were loaded onto double-decked aluminum semi-trailers and transported ~322 km to a commercial abattoir for harvest following a ~ 4- to 5-h lairage. Steers were harvested during the second production shift of each harvest day, starting at approximately 1500 h. The slaughter process was conducted in accordance with USDA-FSIS requirements and standard slaughter site procedures.

Carcass identification was maintained by trained personnel from the Beef Carcass Research Center (BCRC; West Texas A&M University, Canyon, TX). Ear tag numbers were recorded in sequential order and a BCRC carcass tag was placed on the carcass in order to maintain identification and sequence. The processor assigned carcass identification number was correlated to the individual ear tag number and assigned BCRC carcass tag number. During the data collection phase, HCW was recorded following an industry-standard dressing procedure.

Additionally, liver abscess scores (Brown and Lawrence, 2010) were collected on the harvest floor while maintaining sequential identification. Liver abnormalities were observed during harvest and scored as: edible liver; A- = 1–2 small abscesses or inactive scars; A = 1–2 large abscesses or multiple small abscesses; A+ = multiple large abscesses; A + AD = liver adhered to diaphragm; A + OP = open liver abscess; A + AD/OP = adhered to diaphragm with an open liver abscess; cirrhosis, flukes, passive congestive liver, and telangiectasis.

Carcass measurements occurred 26 h after harvest, following the commercial processors standard chilling time. Using the USDA E + V camera system, objective measurements were collected which included marbling score, LMA, and 12^th^ rib fat thickness. Skeletal maturity was obtained only on carcasses designated “B” maturity or greater, otherwise, maturity outcomes were not obtained.

### Striploin Steak Collection

Fifteen carcasses from each pen were identified via random number generator to collect ten striploin steak samples. Five additional carcasses were randomly selected to be used as an alternate, if needed. Carcasses were prescreened for extreme weight outliers, missing identification, or defects. Selected carcasses were railed off-line by trained processor staff for steak collection. A 3.81-cm-thick slice of the longissimus lumborum was collected at the 13^th^ rib of selected carcasses for tenderness evaluation and proximate analysis. Individual steaks were placed into a plastic bag with the appropriate carcass identification tag.

Upon arrival at West Texas A&M University, steaks were vacuumed packaged and aged until 21d postmortem. On the last day of aging, steaks were frozen at −12 °C.

On the designated day for proximate compositional analysis, a 1.27-cm-thick slice was cut using a bandsaw (Biro; Model 3334 Marblehead, OH). The remaining portion of the steak (2.54 cm) was placed back into the freezer for slice shear analysis at a later date.

### Slice Shear Force Analysis

Steaks were randomly assigned a cook order. Steaks were thawed at 2–4 °C for 24 h before cooking. Steaks were cooked to an internal temperature of 71 °C using a forced-air convection oven (model DFG-100-3; Blodgett, Essex Junction, VT) set to 177 °C. Temperature was monitored using a six channel temperature data logger (Omega Engineering, model RDXL6SD-USB Manchester, UK), attached to thermocouple wires (copper-constantan, Type T, Omega Engineering) which were inserted into the geometric center of each uncooked steak to monitor the internal temperature. An initial temperature of the steak prior to cooking was recorded. Individual steaks were weighed before and after cooking to determine cooking loss.

Objective tenderness was assessed according to the Research Guidelines for Cookery, Sensory Evaluation, and Instrumental Tenderness Measurements of Meat (American Meat Science Association, 2016). Immediately after cooking, a 1- to 2-cm slice was removed from the lateral end of each steak parallel to the muscle fiber. Using a sample sizer, a cut was made across the *longissimus* parallel to the first cut at a distance 5 cm medial from the first cut. Using a knife consisting of two parallel blades spaced 1 cm apart, two parallel cuts were simultaneously made through the length of the 5-cm long steak portion at a 45° angle to the long axis of the longissimus and parallel with the muscle fibers. After each slice was obtained, slices were sheared across the muscle fiber using an Instron universal testing machine (model 6800, Norwood, MA) with a crosshead speed of 500 mm·min^−1^ and a 2kN load cell attached to the Slice Shear Force (SSF) blade. Peak shear force values were recorded in kg.

### Intramuscular Fat and Ether Extract Analysis

Striploin cross-section slices were removed from the freezer one at a time for processing. External fat, epimysial connective tissue, and minor muscles were excised from each slice, leaving only the *longissimus dorsi* muscle. Following dissection, each steak was cut into small pieces and submerged into liquid nitrogen until all pieces were completely frozen. Frozen samples were removed using a stainless-steel skimmer and transferred into a Ninja Professional Plus Blender (Model BN700; Shark Ninja LLC USA, Needham, MA). Each sample was pulverized for approximately 10s on low speed (3,700 RPM) and 30s on high speed (5,000 RPM) until a homogenous powder was formed. A 50 g aliquot of the homogenate was obtained for proximate analysis and a 100 g aliquot of the homogenate was retained as a back-up/archive sample. Samples were labeled and placed into a 7.62 × 12.7 cm plastic bag until analysis at SDK Laboratory (Hutchinson, KS).

### Statistical Analysis

Statistical analyses were conducted using the MIXED analysis of variance procedure of SAS (Version 9.4). Data were analyzed as a randomized complete block design. Deads and removals were excluded from analysis of the live performance data. Treatment was the fixed effect, block was a random effect, means were separated with the Tukey-Kramer adjustment, and contrasts were calculated to test linear or quadratic effects of EXP across DOF. Pen was the experimental unit for each outcome. Differences were deemed significant at (*P* ≤ 0.05) for primary discrete variables, with tendencies considered at (*P* ≤ 0.10).

## RESULTS AND DISCUSSION

### Health Performance

Overall health was evaluated in steers during the trial from day 1 to 226 by treatment (Table 2). There was no evidence for a difference between treatments for any measured health outcome during the entire feeding period which included morbidity treatment categories of respiratory, digestive, mechanical and other (*P* ≥ 0.27). Mortality percentages did not differ (*P* = 0.47) across treatments. Mortalities all of categories (respiratory, digestive, mechanical, other) were not different (*P* ≥ 0.13) across treatments. “Other” mortality included heat stress, liver failure, myocarditis, and peritonitis. Morbidity frequencies numerically ranged from 11.71 (CON) to 16.63% (0EXP), and did not differ (*P* ≥ 0.47) across treatments. Frequency of steers retreated ranged from 17.22% (0EXP) to 33.48% (+28EXP), which did not differ (*P* = 0.22) across treatments. All retreat reasons were due to respiratory issues across treatments, except for 2.78% mechanical for +28EXP. Kube et al. (2021) reported animals removed (animals found dead or removed from the treatment phase for health reasons) ranged from 1.5% to 2.5% across treatments. Mortality during pre- and post-treatment initiation was not different across treatments and averaged 1.67% and 0.48%, respectively. Moreover, Teeter et al. (2021) reported mortality being similar across treatments with causes of death being ruminal acidosis as the likely cause of four of the six mortalities. Furthermore, McAtee et al. (2024) reported morbidity, mortality, and total removals to not be different across EXP and OPT treatments.

**Table 2.** Overall health (d 1–d 226) of steers finishing trial by treatment with deads and rejects included.

Item	−14EXP	0EXP	OPT	CON	+14EXP	+28EXP	*P*-value
							Treatment
Head out, n	421	418	419	419	419	421	--
Pens, n	6	6	6	6	6	6	--
Dead, n	2	5	4	9	5	8	--
Mortality, %	0.48	1.19	0.95	2.15	1.18	1.89	0.47
Respiratory, %	0.00	0.48	0.72	0.96	0.94	1.19	0.13
Digestive, %	0.48	0.24	0.24	0.24	0.00	0.24	0.90
Mechanical, %	0.00	0.24	0.00	0.24	0.00	0.00	0.56
Other, %	0.00	0.24	0.00	0.72	0.24	0.47	0.40
Pulled, n	67	70	55	49	62	62	--
Morbidity, %	15.87	16.63	13.16	11.71	14.74	14.69	0.47
Respiratory, %	15.40	15.92	12.68	11.47	14.50	13.97	0.56
Digestive, %	0.00	0.00	0.24	0.00	0.00	0.00	0.44
Mechanical, %	0.00	0.24	0.24	0.00	0.00	0.48	0.65
Other, %	0.47	0.48	0.00	0.24	0.24	0.24	0.74
Retreated, n	22	14	15	15	13	22	--
Retreat, %	28.63	17.22	21.70	30.13	16.43	33.48	0.22
Respiratory, %	28.63	16.63	21.70	30.13	16.43	30.70	0.27
Digestive, %	0.00	0.00	0.00	0.00	0.00	0.00	--
Mechanical, %	0.00	0.60	0.00	0.00	0.00	2.78	0.49
Reject, %	1.90	3.56	1.67	1.91	0.71	2.13	0.33
Buller, %	0.24	0.48	0.24	0.24	0.24	0.24	0.97

^1^14d Experior = 56d treatment followed by 4d withdrawal, 184 d on feed.

^2^0d Experior = 56d treatment followed by 4d withdrawal, 198 d on feed.

^3^Optaflexx = 35d treatment, no withdrawal, 198 d on feed.

^4^Control = no beta agonist treatment.

^5^+14d Experior = 56d treatment followed by 4d withdrawal, 214 d on feed.

^6^+28d Experior = 56d treatment followed by 4d withdrawal, 226 d on feed.

### Live Growth Performance

Feedlot growth performance characteristics are outlined in Table 3. Initial BW did not differ (*P* ≥ 0.38) between EXP treatments; pen mean weights ranged from 333.50 to 338.93 kg. No treatment differences (*P* ≥ 0.72) were observed for (cumulative) daily dry matter intake (DMI). Teeter et al. (2021) also indicated no effect of EXP treatment on DMI (91d period). In contrast, previous results by Kube et al. (2021) reported DMI increased 2–3% (last 60-d period) in EXP-fed steers compared to CON, with EXP doses ranging from 1.5 to 5.5 mg·kg^−1^ DM. Final BW of OPT or 0EXP steers was similar (*P* ≥ 0.38) to CON, however +14EXP tended (*P* = 0.06) to differ and +28EXP steers were 31.26–34.72 kg heavier (*P* < 0.01) than OPT or CON. Teeter et al. (2021) also indicated final BW (with steer and heifer blocks) was not altered by EXP treatment whereas Kube et al. (2021) reported steer final BW increased 11.6–15.7 kg. Feeding OPT resulted in BW changes that are approximately ½ the weighted effect size reported in a meta-analysis by Lean et al. (2014). Compared to CON, live average daily gain (ADG) and gain to feed (G:F) of steers in OPT, 0EXP, +14EXP, and +28EXP treatments were similar (*P* ≥ 0.13). Average daily gain and G:F of the OPT treatment was greater (*P* ≤ 0.01) by 5.2–5.8% and 5.6–6.7%, respectively compared to +14EXP or +28EXP. A linear decrease (*P* < 0.01) was observed with a −0.004 kg·d^−1^ rate of change in ADG for EXP treatments with DOF ranging from 184–226 d. Galyean et al. (2023) reported the effects of extended days on feed while supplementing OPT upon ADG and observed a linear decrease of −0.0029 kg/d. Furthermore, a linear decrease (*P* < 0.01) for EXP treatments was observed with a −0.0004 kg·d^−1^ rate of change in G:F. Similarly, Kube et al. (2021) reported ADG and G:F improved 11.3–15.5% and 9.0–12.0%, respectively, in cattle receiving EXP the final 56 d on feed. In contrast, Teeter et al. (2021) reported EXP fed at doses of 1.38, 5.5, and 22.0 mg·kg^−1^ DM basis the final 91 d on feed tended to differ compared to CON. McAtee et al. (2024) reported ADG and G:F improved by 2.96% and 6.59% when comparing EXP to OPT. Furthermore, the variance in responses across other trials with EXP supplementation may be due to differences in DOF of the trial and different start dates for the trial. Moreover, the typical OPT responses in ADG and G:F reported by Lean et al. (2014) was not observed in this trial, rather the OPT response was muted.

**Table 3.** Least square means for the effect of Experior on growth performance traits over a 56-d treatment period (deads and rejects excluded).

Item	−14EXP^1^	0EXP^2^	OPT^3^	CON^4^	+14EXP^5^	+28EXP^6^	SEM	*P*-value		
								Treatment	Linear	Quadratic
DOF	184	198	198	198	214	226	--	--	--	--
Initial BW, kg	333.50	336.85	334.50	338.70	338.60	338.93	3.28	0.38	--	--
Final BW, kg	655.53^c^	680.62^b^	673.83^b^	670.37^b c^	685.87^b^	705.09^a^	4.74	<0.01	<0.01	0.43
Total gain, kg	322.03^d^	343.77^bc^	339.33^b,c^	331.67^c^^d^	347.27^b^	366.16^a^	5.59	<0.01	<0.01	0.69
DMI, kg	9.73	9.83	9.67	9.73	9.66	9.76	0.09	0.72	--	--
ADG, kg	1.75^a^	1.74^a,b^	1.72^a,b^	1.68^b,c^	1.63^c^	1.62^c^	0.02	<0.01	<0.01	0.73
G:F	0.180^a^	0.177^a,b^	0.178^a,b^	0.172^b,c^	0.168^c^	0.166^c^	0.002	<0.01	<0.01	0.64
Carcass ADG^7^, kg	1.28^a^	1.25^a,b^	1.21^b,c^	1.17^c^	1.21^b,c^	1.19^c^	0.01	<0.01	<0.01	0.88
Carcass G:F^7,^ kg	0.131^a^	0.127^a,b^	0.125^b,c^	0.121^c^	0.126^b,c^	0.122^c^	0.001	<0.01	<0.01	0.83

^1^−14d Experior = 56d treatment followed by 4d withdrawal, 184 d on feed.

^2^0d Experior = 56d treatment followed by 4d withdrawal, 198 d on feed.

^3^Optaflexx = 35d treatment, no withdrawal, 198 d on feed.

^4^Control = no beta agonist treatment.

^5^+14d Experior = 56d treatment followed by 4d withdrawal, 214 d on feed.

^6^+28d Experior = 56d treatment followed by 4d withdrawal, 226 d on feed.

^7^Estimated initial hot carcass weight using calculations described by Tatum et al. (2012) as HCW = 0.2598 x Initial BW^1.1378^.

^a, b, c^Within a parameter, means with differing superscripts differ (*P* < 0.05).

For EXP treatments, HCW responses improved relative to CON and OPT, adjustments in carcass-based ADG and G:F, along with corresponding rises in carcass-based cost of gain, exhibit a less abrupt change during the later phase of the finishing period compared to changes in similar performance indictors based on body weight (Tatum et al., 2012). Carcass ADG of 0EXP steers was 6.8% greater (*P* < 0.01) than CON, which had similar (*P* ≥ 0.10) carcass ADG to OPT, +14EXP, and +28EXP steers. A linear decrease (*P* < 0.01) for EXP treatments was observed with a −0.002 kg·d^−1^ rate change in carcass ADG. Carcass gain to feed of 0EXP steers was improved 5.0% (*P* = 0.01) compared to CON, which declined (*P* = 0.05) to 4.1% for +14EXP steers compared to CON. Furthermore, a linear decrease (*P* < 0.01) for EXP treatments with added total DOF was observed with a −0.0002 kg·d^−1^ rate of change in G:F.

### Calculated Cumulative Ammonia Gas Emissions

Calculated cumulative ammonia gas emissions (CCAGE) expressed either as total NH_3_ emitted (g) or NH_3_ emitted per kg of live or hot carcass weight was reduced (*P* < 0.01) by 5.6–8.5%, 5.1–10.7%, and 6.3–13.6%, respectively for 0EXP, +14EXP and +28EXP compared to CON (Table 4). Cattle fed EXP experienced a 5.1–6.3% (170–212 g) reduction in CCAGE when compared to CON cattle (*P* < 0.01). Compared to CON and to each EXP treatment, the reduction in CCAGE when standardized by HCW or BW were greater (*P* < 0.01) as DOF were added for 0EXP, +14EXP, and +28EXP steers. Results by Kube et al. (2021) suggested that as lubabegron dose increased, CCAGE standardized by BW and HCW were reduced. Furthermore, Kube et al. (2021) estimated lubabegron reduced NH_3_ gas emissions per unit of HCW by 3.8–14.6% when fed at 1.5–5.5 mg of lubabegron·kg^−1^ of DM for 56 d. More recently, Vogel et al. (2023) estimated CCAGE to be reduced by 14.6% in steers fed EXP the final 56 d on feed. Calculation of CCAGE for the OPT treatment was not possible because the Brown et al. (2019) equation does not provide a slope for OPT. However, a study by Ross et al. (2021) examining ractopamine hydrochloride (RAC) in finishing steers reported that feeding RAC resulted in a 17.21% reduction in NH_3_ emissions from day 0 to 28 and a tendency for a reduction of 11.07% by day 42 compared to control (CON).

**Table 4.** Least square means for the effect of Experior on calculated cumulative ammonia emissions^7^ during a 56-d treatment period.

Item	−14EXP^1^	0EXP^2^	OPT^3^	CON^4^	+14EXP^5^	+28EXP^6^	SEM	*P*- value		
								Treatment	Linear	Quadratic
Cumulative NH_3_ gas emissions, g	3223.39^a,b^	3150.53^b^	--	3338.01^a^	3167.56^b^	3126.23^b^	41.12	<0.01	0.05	0.60
Cumulative NH_3_ gas emissions, g/kg BW	4.92^a^	4.63^b^	--	4.98^a^	4.62^b^	4.43^c^	0.04	<0.01	<0.01	0.07
Cumulative NH_3_ gas emissions g/kg HCW	7.55^b^	7.13^c^	--	7.79^a^	6.96^c^	6.73^d^	0.07	<0.01	<0.01	0.05

^1^−14d Experior = 56d treatment followed by 4d withdrawal, 184 d on feed.

^2^0d Experior = 56d treatment followed by 4d withdrawal, 198 d on feed.

^3^Optaflexx = 35d treatment, no withdrawal, 198 d on feed.

^4^Control = no beta agonist treatment.

^5^+14d Experior = 56d treatment followed by 4d withdrawal, 214 d on feed.

^6^+28d Experior = 56d treatment followed by 4d withdrawal, 226 d on feed.

^a, b, c^Within a parameter, means with differing superscripts differ (*P* < 0.05).

^7^Determined ammonia gas emissions using calculations described by Brown et al. (2019) as y [log10(cumulative ammonia gas emissions, g/animal)] = 0.06758372 × (cumulative N intake, kg/animal) − 0.011425 × (lubabegron dose, g/ton) − 4.9743281 × (1/cumulative duration in days) − 0.0012361 × (cumulative N intake, kg/animal)2 + 0.0002744 × (indoor temperature, °C)2 + 3.01996229.

A pivotal 91-d study demonstrated the clinical effectiveness of lubabegron at doses as low as 1.5 mg of LUB·kg^−1^ of DM in reducing NH_3_ gas emissions·kg^−1^ BW and HCW (FDA-FOI, 2018; Teeter et al., 2021). Approximately 10–30% of dietary nitrogen (N) is utilized by the animal and deposited as protein in tissues, with the remaining N emitted into the environment through fecal and urinary excretion, where it becomes susceptible to volatilization as NH_3_ (Cole et al., 2006). Furthermore, decreasing ammonia gas emissions can mitigate the adverse effects on water quality by reducing eutrophication and acidification, as well as air quality by minimizing the formation of fine particles (OECD, 2013). Supplementation of lubabegron reduces NH_3_ gas emissions per unit of BW and HCW, which implies improved N retention.

### Mobility Scores

The percentage of cattle with normal mobility (i.e., mobility score = 1) was not different (*P* ≥ 0.17) for OPT, −14EXP, and 0EXP compared to CON (Table 5). However, the percentage of cattle with normal mobility (i.e., mobility score = 1) was lower (*P* < 0.05) for +14EXP and +28EXP compared to OPT or CON. Frequency of mobility scores for cattle scoring a 2 were higher (*P* ≤ 0.02) for +14EXP and +28EXP compared to 0EXP and CON. Mobility scores of 2 were assigned when cattle exhibited stiffness, shortened stride, or slight limp. As the cattle became heavier and were on feed for longer days, there was an increased mobility risk potential. Animals receiving abnormal scores of 3 were in greater proportion (*P* = 0.03) for −14EXP when compared to OPT and CON. However, overall animals receiving abnormal scores of 3 were not different (*P* ≥ 0.37) between 0EXP, +14EXP, +28EXP, OPT and CON. A mobility score of 4 or the occurrence of a downer animal was never observed. Kube et al. (2021) reported animals receiving abnormal scores of > 2 were not different across treatments. McAtee et al. (2024) results tended to differ in cattle fed EXP, whereas Kube et al. (2021) and Vogel et al. (2023) reported no differences in mobility score of cattle fed EXP compared to control.

**Table 5.** Animal mobility scores prior to harvest of steers fed Experior for the final 56 d of the feeding period.

Mobility	−14EXP^1^	0EXP^2^	OPT^3^	CON^4^	+14EXP^5^	+28EXP^6^	SEM	*P*- value		
								Treatment	Linear	Quadratic
DOF	184	198	198	198	214	226	--	--	--	--
Score 1, %	94.15^a,b^	95.43^a,b^	95.83^a^	96.28^a^	93.18^b^	93.06^b^	0.65	<0.01	0.05	0.26
Score 2, %	4.15^b,c^	3.56^c^	4.17^a,b,c^	3.72^c^	6.33^a,b^	6.46^a^	0.53	<0.01	<0.01	0.50
Score 3, %	1.70^a^	1.02^a,b^	0.00^b^	0.00^b^	0.49^a,b^	0.48^a,b^	0.39	0.02	0.02	0.36
Score 4, %	0.00	0.00	0.00	0.00	0.00	0.00	--	--	--	--
Downer, %	0.00	0.00	0.00	0.00	0.00	0.00	--	--	--	--

^1^−14d Experior = 56d treatment followed by 4d withdrawal, 184 d on feed.

^2^0d Experior = 56d treatment followed by 4d withdrawal, 198 d on feed.

^3^Optaflexx = 35d treatment, no withdrawal, 198 d on feed.

^4^Control = no beta agonist treatment.

^5^+14d Experior = 56d treatment followed by 4d withdrawal, 214 d on feed.

^6^+28d Experior = 56d treatment followed by 4d withdrawal, 226 d on feed.

^7^Cattle mobility was scored according the North American Meat Institute (NAMI, 2016). Score 1 = Normal, walks easily with no apparent lameness or change in gait; Score 2 = Keeps up with normal cattle when the group is walking; will exhibit one or more of the following: stiffness, shortness of stride, or slight limp; Score 3 = Animal that lags behind normal cattle when the group is walking; will exhibit one or more of the following: obvious stiffness, difficulty taking steps, obvious limp, or exhibiting obvious discomfort; Score 4 = Extremely reluctant to move even when encouraged by a handler, statue-like.

^a, b, c^Within a parameter, means with differing superscripts differ (*P* < 0.05).

### Liver Outcomes

Liver abscess outcomes were categorized into minor (A- and A) and major (A+, A + AD, A + OP, and A + AD/OP). Liver abscess rates ranged (Table 6) from 16.06% (0EXP) to 21.69% (+28EXP) but did not differ between treatments (*P* = 0.88) and were within normal ranges for feedlot cattle (Amachawadi and Nagaraja, 2016)

**Table 6.** Liver abscess outcomes of steers fed Experior for the final 56 d of the feeding period.

Item	−14EXP^1^	0EXP^2^	OPT^3^	CON^4^	+14EXP^5^	+28EXP^6^	SEM	*P*- value
								Treatment
Edible, %	67.84	75.96	72.72	75.54	70.31	67.99	4.03	0.54
Minor^7^, %	9.98	9.29	10.00	10.90	11.49	9.61	2.07	0.97
Major^8^, %	10.26	6.77	10.38	7.64	9.72	12.08	2.64	0.64
Total Abscess, %	20.24	16.06	20.37	18.54	21.21	21.69	4.04	0.88

^1^−14d Experior = 56d treatment followed by 4d withdrawal, 184 d on feed.

^2^0d Experior = 56d treatment followed by 4d withdrawal, 198 d on feed.

^3^Optaflexx = 35d treatment, no withdrawal, 198 d on feed.

^4^Control = no beta agonist treatment.

^5^+14d Experior = 56d treatment followed by 4d withdrawal, 214 d on feed.

^6^+28d Experior = 56d treatment followed by 4d withdrawal, 226 d on feed.

^7^Minor = A- and A liver abscess scores.

^8^Major = A+, A + Adhesion, A + Open, and A + Adhesion/Open liver abscess scores.

^a, b, c^Within a parameter, means with differing superscripts differ (*P* < 0.05).

### Carcass Outcomes—E + V Camera

Hot carcass weight of 0EXP, +14EXP, or +28EXP steers was 14.32–36.87 kg heavier (*P* < 0.01) than CON, which did not differ (*P* ≥ 0.81) from OPT or −14EXP (Table 7). Hot carcass weight increased linearly (*P* < 0.01) at a rate of +0.91 kg·d^−1^ following EXP supplementation across DOF. Other studies reported HCW to be 11.3–18.0 kg greater (Kube et al., 2021; Vogel et al., 2023) when EXP was supplemented for 56 d. A more recent study conducted by McAtee et al. (2024) reported HCW to be 11 kg heavier when comparing EXP to OPT. The difference between CON and OPT treatments for HCW was lesser than the weighted mean difference reported by Lean et al. (2014) yet was within the range of outcomes reported in that meta-analysis. Linear responses for EXP treatments across different DOF were observed for dressing percentage (*P* < 0.01), LMA:HCW (*P* < 0.01), backfat thickness (*P* < 0.01), marbling score (*P* = 0.02), yield grade (*P* < 0.01), and empty body fat (*P* < 0.01; EBF). Galyean et al. (2023) investigated the impact of extended days on feed while supplementing OPT and observed a linear increase of 0.0303 for dressing percentage, 0.7862 for HCW, 0.0033 for twelfth-rib backfat thickness, 0.0955 for LM area, and a linear increase in marbling score by 0.0569. Additionally, Galyean et al. (2023) reported zilpaterol hydrochloride supplementation and observed a linear increase of 0.0249 for dressing percentage, 0.9184 for HCW, 0.0032 for LM area, and a linear increase in marbling score by 0.1030. Steers fed EXP had 0.9–2.5% point increase (*P* < 0.01) in dressed yields. Previous EXP studies reported steers supplemented with lubabegron had greater dressing percentages and LMA compared to CON (Kube et al., 2021; Vogel et al., 2023). Twelfth-rib backfat was similar (*P* ≥ 0.86) for 0EXP and +14EXP, and greater (*P* = 0.03; 1.76 vs. 1.91 cm^2^) for +28EXP compared to CON. A linear increase (*P* < 0.01) was observed with a +0.007 d^−1^ rate of change in twelfth-rib backfat for EXP treatments with DOF ranging from 184–226 d. Longissimus muscle area was 4.03–6.23 cm^2^ larger (*P* < 0.01) in steers fed EXP compared to CON, which tended to differ (*P* = 0.08) from OPT. Carcasses in the OPT treatment had LMA growth and subtle lowering of backfat in comparison to the CON treatment consistent with meta-analysis outcomes reported by Lean et al. (2014). Longissimus muscle area was 6.2 cm^2^ larger (*P* < 0.01) for +14EXP and +28EXP compared to CON. Marbling score was lower (*P* ≤ 0.02) for −14EXP than CON or OPT, tended (*P* = 0.09) to be lower for 0EXP than CON, and was similar (*P* ≥ 0.28) for + 14EXP and +28EXP compared to CON and OPT with carcasses of EXP fed steers having marbling scores 14–39 points less than CON carcasses. Teeter et al. (2021) reported marbling score tended to differ with scores 50–63 points less, compared to CON. Calculated YG did not differ (*P* ≥ 0.42) for 0EXP, +14EXP, +28EXP and OPT, however −14EXP carcasses had numerically lower yield grades (*P* < 0.01) compared to CON. As expected, calculated YG was numerically the lowest (3.08) for −14EXP due to steers being on feed the fewest days. Weight corrected LMA (LMA:HCW) was 1.33 cm^2^ · 100 kg^−1^ larger (*P* < 0.01) for −14EXP compared to CON. Weight corrected LMA was similar (*P* ≥ 0.70) for 0EXP and +14EXP compared to OPT and CON. Calculated empty body fat percentage did not differ (*P* ≥ 0.15) for 0EXP, +14EXP, +28EXP and OPT compared to CON. However, EBF for −14EXP was lower (*P* < 0.01) compared to CON.

**Table 7.** Least square means of E + V camera assessed carcass outcomes for steers fed Experior for the final 56 d of the feeding period.

Item	−14 EXP^1^	0 EXP^2^	OPT^3^	CON^4^	+14 EXP^5^	+28 EXP^6^	SEM	*P—*value		
								Treatment	Linear	Quadratic
Hot Carcass Weight, kg	426.27^d^	441.82^c^	431.25^d^	427.50^d^	454.47^b^	464.37^a^	2.95	<0.01	<0.01	0.20
Dressed Yield, %	65.04^b^	64.93^b^	64.01^c^	63.78^c^	66.28^a^	65.87^a^	0.17	<0.01	<0.01	0.25
LMA:HCW, cm^2^ · 100 kg^−1^	24.00^a^	22.84^b,c^	23.00^b^	22.67^b,c^	22.70^b,c^	22.21^c^	0.17	<0.01	<0.01	0.05
Backfat, cm	1.60^c^	1.71^b,c^	1.73^bc^	1.76^b^	1.80^a,b^	1.91^a^	0.03	<0.01	<0.01	0.96
LM Area, cm^2^	102.29^a^	100.93^ab^	99.21^b,c^	96.90^c^	103.13^a^	103.11^a^	0.75	<0.01	0.08	0.24
Marbling^7^	459^b^	474^a,b^	490^a^	498^a^	484^a,b^	479^a,b^	7.11	<0.01	0.02	0.14
Yield Grade	3.08^c^	3.38^b^	3.40^b^	3.51^a,b^	3.48^a,b^	3.67^a^	0.06	<0.01	<0.01	0.35
EBF^8^, %	31.12^c^	32.25^b^	32.39^b^	32.69^a,b^	32.88^a,b^	33.46^a^	0.23	<0.01	<0.01	0.22

^1^−14d Experior = 56d treatment followed by 4d withdrawal, 184 d on feed.

^2^0d Experior = 56d treatment followed by 4d withdrawal, 198 d on feed.

^3^Optaflexx = 35d treatment, no withdrawal, 198 d on feed.

^4^Control = no beta agonist treatment.

^5^+14d Experior = 56d treatment followed by 4d withdrawal, 214 d on feed.

^6^+28d Experior = 56d treatment followed by 4d withdrawal, 226 d on feed.

^7^Marbling Score: 300 = Slight^0^; 400 = Small^0^; 500 = Modest^0^.

^8^Estimated empty body fat using calculations described by Guiroy et al. (2001) as EBF = 17.76207 + (4.6812 × fat thickness) + (0.01945 × HCW) + (0.81855 × quality grade) − (0.06754 × ribeye area).

^a, b, c^Within a parameter, means with differing superscripts differ (*P* < 0.05).

### USDA Yield Grade and Quality Grade

The USDA stamped carcass quality grade (QG) frequency of Prime, Upper 2/3 Choice, Low Choice, and Select (Table 8) were reported. Proportion of stamped Prime QG did not differ between treatments (*P* = 0.24). Cattle on feed for 214 d or 228 d (+14EXP; +28EXP) had a QG of 3.69% and 3.67% Prime whereas −14EXP cattle were only 0.98% Prime.

**Table 8.** E + V Camera assessed quality and yield outcomes of steers fed Experior for the final 56 d of the feeding period.

Item	−14EXP^1^	0EXP^2^	OPT^3^	CON^4^	+14EXP^5^	+28EXP^6^	SEM	*P*- value		
								Treatment	Linear	Quadratic
Prime, %	0.98	2.56	1.21	1.99	3.69	3.67	0.98	0.24	--	--
Upper 2/3 Choice, %	22.02^c^	28.39^b,c^	40.54^a^	38.01^a,b^	29.87^a,b,c^	27.06^b,c^	2.92	<0.01	0.17	0.09
Low Choice, %	61.13^a^	50.54^a,b^	48.17^b^	50.07^a,b^	52.05^a,b^	54.92^a,b^	2.56	0.02	0.15	0.01
Select, %	15.87^a,b^	18.50^a^	10.09^b^	9.93^b^	14.40^a,b^	14.35^a,b^	2.23	0.01	0.28	0.45
YG 1, %	10.96^a^	6.33^a,b^	4.18^b^	4.23^b^	4.91^b^	3.70^b^	1.34	<0.01	<0.01	0.21
YG 2, %	36.35^a^	26.73^a,b^	29.93^a,b^	25.15^a,b^	26.42^a,b^	19.79^b^	2.77	<0.01	<0.01	0.58
YG 3, %	41.38	44.63	41.47	42.03	42.25	43.12	3.18	0.98	--	--
YG 4, %	9.08^b^	18.77^a^	21.48^a^	24.58^a^	20.29^a^	24.97^a^	2.21	<0.01	<0.01	0.26
YG 5, %	2.23^b^	3.55^b^	2.95^b^	4.03^b^	6.13^a,b^	8.42^a^	0.99	<0.01	<0.01	0.62
Carcasses 454–475.9 kg, %	16.61	19.36	19.64	16.96	19.25	20.82	1.72	0.37	--	--
Carcasses 476–498.4 kg, %	5.37^b^	13.25^a,b^	6.64^b^	6.19^b^	15.82^a^	17.77^a^	1.97	<0.01	<0.01	0.14
Carcasses ≥ 498.5 kg, %	3.92^b^	9.15^b^	5.11^b^	3.50^b^	18.06^a^	23.38^a^	1.82	<0.01	<0.01	0.98
Over 30 months, %	4.35	5.56	5.86	6.00	8.55	8.56	1.93	0.09	0.01	0.70

^1^−14d Experior = 56d treatment followed by 4d withdrawal, 184 d on feed.

^2^0d Experior = 56d treatment followed by 4d withdrawal, 198 d on feed.

^3^Optaflexx = 35d treatment, no withdrawal, 198 d on feed.

^4^Control = no beta agonist treatment.

^5^+14d Experior = 56d treatment followed by 4d withdrawal, 214 d on feed.

^6^+28d Experior = 56d treatment followed by 4d withdrawal, 226 d on feed.

^a, b, c^Within a parameter, means with differing superscripts differ (*P* < 0.05).

Proportion of stamped Upper 2/3 Choice QG was the highest for OPT, which differed (*P* ≤ 0.03) from −14EXP and 0EXP. Additionally, the proportion of stamped Upper 2/3 Choice QG for 0EXP, + 14EXP, and OPT were similar (*P* ≥ 0.14) when compared to CON. Frequency of Low Choice for 0EXP, +14EXP, +28 EXP, and OPT was similar (*P* ≥ 0.76) to CON. However, −14EXP tended (*P* = 0.053) to differ from CON, when comparing frequency of Low Choice. Compared to CON, the frequency of stamped Select QG was greater (*P* = 0.02) for 0EXP, however −14EXP, +14EXP, 28EXP, and OPT were similar (*P* ≥ 0.20) to CON. The mode of action of lubabegron is not lipolytic, however rapidly increasing longissimus muscle area dilutes marbling, thus lowering quality grades for EXP treatments. At the same DOF, previous studies have reported reduced quality by lessening marbling (Kube et al., 2021; Teeter et al., 2021; Vogel et al., 2023; McAtee et al., 2024) as a result of lubabegron supplementation. Frequency of steers stamped yield grade (YG) 1 was greater (*P* = 0.01) for −14EXP compared to CON, however 0EXP, +14EXP, +28EXP and OPT did not differ (*P* ≥ 0.86) compared to CON. Frequency of steers stamped YG 2 were similar (*P* ≥ 0.71) for 0EXP, +14EXP, +28EXP, and OPT, but −14EXP tended (*P* = 0.06) to be greater than CON. The yield grade distribution shifted numerically leaner with EXP and less DOF. Frequency of steers stamped YG 3 was similar (*P* ≥ 0.98) across all treatments. Compared to CON, the frequency of steers stamped YG 4 was similar (*P* ≥ 0.43) for 0EXP, +14EXP, +28EXP and OPT. In contrast −14EXP cattle were 9.08% YG 4 carcasses and differed (*P* ≤ 0.04) from 0EXP, +14EXP, +28EXP, OPT and CON. Frequency of steers stamped YG 5 was larger (*P* = 0.04) for +28EXP compared to CON. Similar findings by Kube et al. (2021) illustrated more YG 2 carcasses and fewer YG 4 carcasses when steers were supplemented with EXP compared to CON.

### Heavy Carcasses and Carcasses of 30 Months of Age

Frequency of carcasses weighing 454–475.9 kg did not differ (*P* = 0.37) between treatments. Numerically, the frequency of cattle with HCW between 454 to 475.9 kg, ranged from 16.61% (−14EXP) to 20.82% (+28EXP). As DOF were added, the frequency of HCW between 476 to 498.4 kg increased (*P* ≤ 0.03) for +14EXP and +28EXP as compared to CON or OPT. Carcasses ≥ 498.5 kg were greater in frequency (*P* < 0.01) for +14EXP and +28EXP at 18.06% and 23.38%, respectively than all other treatments. Carcasses over 30 mo of age ranged from 4.35% (−14EXP) to 8.56% (+28EXP) across treatments and tended (*P* = 0.09) to increase with added DOF.

### Slice Shear Force

**Figure 1.**
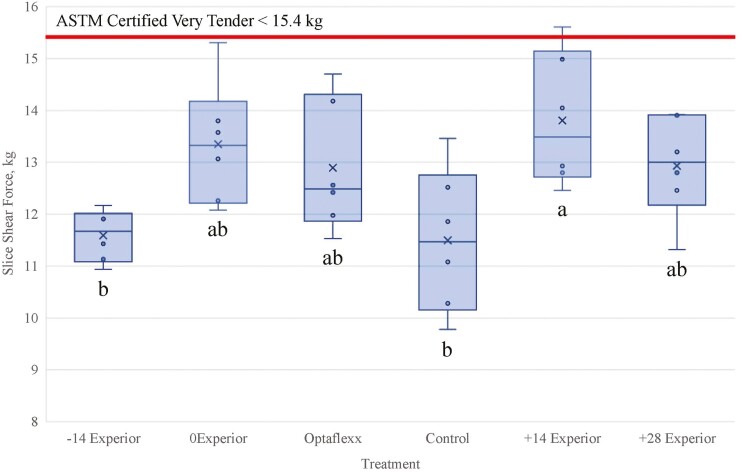
Least square means of *longissimus dorsi* slice shear force outcomes of steers fed Experior for the final 56-d of the feeding period. Treatments identified as: −14d Experior = 56d treatment followed by 4d withdrawal, 184 d on feed; 0d Experior = 56d treatment followed by 4d withdrawal, 198 d on feed; Optaflexx = 35d treatment, no withdrawal, 198 d on feed; Control = no beta agonist treatment; +14d Experior = 56d treatment followed by 4d withdrawal, 214 d on feed; +28d Experior = 56d treatment followed by 4d withdrawal, 226 d on feed. Means with differing superscripts differ (*P* < 0.05).

Slice shear force values of a subset (n = 361) of total cattle were 20.1% greater (*P* = 0.02) for +14EXP compared to CON, whereas 0EXP tended (*P* = 0.09; 16.1%) to be greater and +28EXP, −14EXP and OPT were similar (*P* ≥ 0.28) to CON (Figure 1). Slice shear force means for each treatment were 11.59 kg (−14EXP), 13.35 kg (0EXP), 12.90 kg (OPT), 11.50 kg (CON), 13.81 kg (+14EXP), and 12.94 kg (+28EXP). The subtle increase in SSF of the OPT treatment over CON is consistent with the meta-analysis reported by Lean et al. (2014). As described by ASTM (2011), a tenderness marketing claim is available for labeling and advertisements associated with beef cuts in order to promote and distinguish a premium marketplace. According to the ASTM (2011) SSF thresholds, certified tender *longissimus* is < 20 kg and certified very tender is < 15.4 kg. The percentage of certified very tender steaks was 93% (−14EXP), 78% (0EXP), 79% (OPT), 93% (CON), 63% (+14EXP), and 80% (+28EXP). Corona (2020) reported that as the feeding duration of lubabegron increased from 28 to 84 d, SSF values became tougher; however, the 56 d treatment resulted in SSF values being similar to those fed 28 and 84 d. Additionally, Hays et al. (2023) reported lubabegron supplemented steers had a higher SSF value at early aging timepoints.

### Intramuscular Fat Analysis

**Figure 2.**
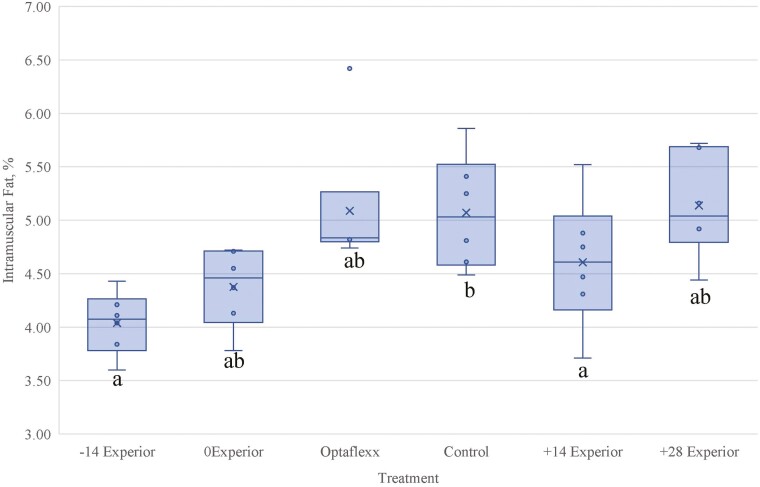
Least square means of *longissimus dorsi* intramuscular fat content of steers fed Experior for the final 56-d of the feeding period. Treatments identified as: −14d Experior = 56d treatment followed by 4d withdrawal, 184 d on feed; 0d Experior = 56d treatment followed by 4d withdrawal, 198 d on feed; Optaflexx = 35d treatment, no withdrawal, 198 d on feed; Control = no beta agonist treatment; +14d Experior = 56d treatment followed by 4d withdrawal, 214 d on feed; +28d Experior = 56d treatment followed by 4d withdrawal, 226 d on feed. Means with differing superscripts differ (*P* < 0.05).

The intramuscular fat (IMF) percentage determined by ether extract was similar (*P* ≥ 0.44) for +14EXP, +28EXP, and OPT compared to CON, whereas 0EXP tended (*P* = 0.09) to be lower than CON (Figure 2). Numerically, the greatest treatment mean IMF value was 5.14% for +28EXP whereas the lowest treatment mean IMF value was 4.04% for −14EXP. Savell et al. (1986) categorized mean ether extractable fat percentages for marbling levels, reporting a marbling score of Small to represent an IMF value of 4.99%, whereas percentages for Modest and Moderate were 5.97% and 7.34%, respectively. For every 1% increase in IMF percentage, a 31.3 degree increase in marbling score occurred, suggesting a moderate correlation (r = 0.66) between marbling score and IMF percentage.

## CONCLUSION

Experior is a novel tool approved to reduce ammonia emissions per unit of production. In this study, LUB decreased CCAGE and increased HCW with minimal quality changes when fed the last 56 d on feed. At the same DOF, EXP improved carcass performance, HCW, and lowered quality grade distribution with no observed detriment to animal health or wellbeing. Added days on feed diminished live growth performance, resulted in improved marbling, a fatter endpoint, and increased risk of carcass discounts. While feeding EXP compared to OPT resulted in a lower marbling score, EXP outperformed OPT in terms of total kg of HCW.
